# HPV16 E7 Nucleotide Variants Found in Cancer-Free Subjects Affect E7 Protein Expression and Transformation

**DOI:** 10.3390/cancers14194895

**Published:** 2022-10-06

**Authors:** Hong Lou, Joseph F. Boland, Hongchuan Li, Robert Burk, Meredith Yeager, Stephen K. Anderson, Nicolas Wentzensen, Mark Schiffman, Lisa Mirabello, Michael Dean

**Affiliations:** 1 Cancer Genetics Research Laboratory, Division of Cancer Epidemiology and Genetics, Frederick National Laboratory for Cancer Research, Rockville, MD 20850, USA; 2 Basic Science Program, Frederick National Laboratory for Cancer Research, Frederick, MD 21702, USA; 3 Departments of Pediatrics, Microbiology and Immunology, and Obstetrics & Gynecology and Women’s Health, Albert Einstein College of Medicine, Bronx, NY 10461, USA; 4 Laboratory of Cancer Genetics, Division of Cancer Epidemiology and Genetics, National Cancer Institute, Rockville, MD 20850, USA; 5 Laboratory of Translational Genomics, Division of Cancer Epidemiology and Genetics, National Cancer Institute, Rockville, MD 20850, USA

**Keywords:** HPV16, E7 variants, cervical cancer, transformation, wound healing, Western blotting

## Abstract

**Simple Summary:**

Human papillomaviruses (HPV) are one of the deadliest viruses causing cancer resulting in over 300,000 deaths annually in the world. HPV16 is the most oncogenic form of HPV and is responsible for over half of all HPV-driven tumors. The viral E7 gene encodes a powerful oncoprotein that can cause infected cells to have uncontrolled growth. A previous study showed that HPV16 isolates with amino acid alterations in the E7 gene were nearly always found in women with HPV infection but no cancer. To study the role of these variants, we expressed them in cultured cells. Most variants produced lower levels of protein, and selected variants had reduced activity in allowing cells to grow in assays that measure tumor cell invasiveness. The data indicate that inhibition of E7 could be a practical approach to treating HPV-driven cancers.

**Abstract:**

The human papillomavirus (HPV) type 16 E7 oncogene is critical to carcinogenesis and highly conserved. Previous studies identified a preponderance of non-synonymous E7 variants amongst HPV16-positive cancer-free controls compared to those with cervical cancer. To investigate the function of E7 variants, we constructed full-length HPV16 E7 genes and tested variants at positions H9R, D21N, N29S, E33K, T56I, D62N, S63F, S63P, T64M, E80K, D81N, P92L, and P92S (found only in controls); D14E, N29H cervical intraepithelial neoplasia (CIN2), and P6L, H51N, R77S (CIN3). We determined the steady-state level of cytoplasmic and nuclear HPV16 E7 protein. All variants from controls showed a reduced level of E7 protein, with 7/13 variants having lower protein levels. In contrast, 2/3 variants from the CIN3 precancer group had near-wild type E7 levels. We assayed the activity of representative variants in stably transfected NIH3T3 cells. The H9R, E33K, P92L, and P92S variants found in control subjects had lower transforming activity than D14E and N29H variants (CIN2), and the R77S (CIN3) had activity only slightly reduced from wild-type E7. In addition, R77S and WT E7 caused increased migration of NIH3T3 cells in a wound-healing assay compared with H9R, E33K, P92L, and P92S (controls) and D14E (CIN2). These data provide evidence that the E7 variants found in HPV16-positive cancer-free women are partially defective for transformation and cell migration, further demonstrating the importance of fully active E7 in cancer development.

## 1. Introduction

Cervical cancer is the second most common cancer among women worldwide and remains a clinical problem despite early detection and therapy [1]. HPV16 is the most oncogenic HPV type and accounts for 50–70% of cervical cancer. HPV encodes two oncoproteins, E6 and E7 that contribute to cell transformation through disruption of cell cycle regulation and inhibition of apoptosis [2]. The HPV16 E7 oncoprotein is essential to the viral life cycle and throughout the carcinogenic process, from benign precursor lesions to invasive carcinoma [2]. HPV-induced carcinogenesis results from the deregulated expression of the HPV E6 and E7 oncoproteins and E6 and E7 function to establish and maintain a cellular environment that supports viral genome replication [3]. HPV does not encode enzymes for DNA replication; thus, viral genome synthesis uses cellular DNA replication enzymes. Aberrant cell proliferation is a crucial feature of cervical cancer. Cell cycle control is often disturbed; indeed, the p53 and pRB (retinoblastoma protein) tumor suppressor pathways are dysfunctional in most cervical tumors [4,5].

HPV16 E7 is a multifunctional protein interacting with multiple cellular factors [6,7,8,9,10]. One of the main activities of E7 is to induce terminally differentiated cells to re-enter the cell cycle through disruption of the pRB/E2F system. Thus, E7 binds to and targets pRB, which has an essential role in proliferation control by regulating the E2F transcription factor family affecting cell cycle progression, nucleotide synthesis, DNA replication, and apoptosis [11,12].

**Figure 1:**
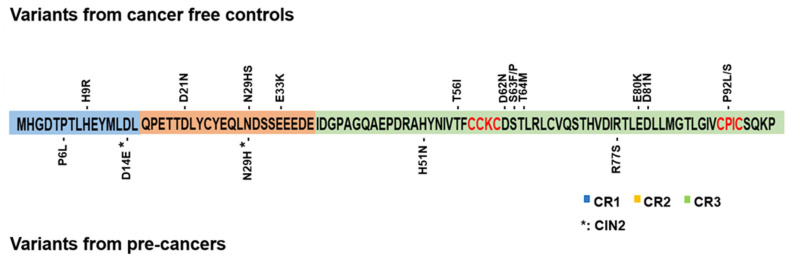
HPV16 E7 variants examined in this study. Schematic representation of amino acid (AA) sequences of HPV16 E7 single AA substitution mutants and their location in functional domains. Variants from cancer-free controls shown above and variants from pre-cancer samples below the functional domain. The numbers 1 to number 98 indicate the amino acid position of E7 variants. *: cervical intraepithelial neoplasia 2 (CIN2); the CXXC motif is shown in red (AA.58–61 and 91–94). Conserved regions 1, 2 and 3 are indicated (CR1, CR2, CR3).

HPV16 E7 is only 98-amino acids, yet it binds to multiple cellular proteins [13]. The E7 protein consists of a C-terminal zinc finger-like domain (residues 38–98), a conserved region 3 (CR3) [14], and N-terminus (residues 1–37), CR1 (residues 1–15) and CR2 (residues 16–37) [14] domains (Figure 1). The CR1 and CR2 domains contain the binding motif for pRB [15]. CR3 has two Cys-X-X-Cys motifs (CXXC) involved in binding a zinc molecule. Point mutations in one of the two CXXC motifs of E7 severely impair the ability to transform rat embryo fibroblasts [16,17,18,19].

In a study of 5570 HPV16 infected cervical precancer/cancer cases and HPV16-positive cancer-free controls, we observed significantly more E7 amino-acid altering variants in controls than in cases [20]. The substitutions are located in multiple domains of the E7 protein, and we hypothesized that the variants do not support carcinogenesis. To test the ability of these variants to affect E7 function, we expressed the variants in multiple in vitro systems to determine steady-state protein expression, transformation, and migration ability.

## 2. Materials and Methods

### 2.1. Plasmid Construction and antiHPV16 E7 Antibodies

A synthetic full-length HPV16 A1 sequence (GenBank accession number is NC-001526.4) was used as a template for preparing an HPV16 E7 construct with the primer set: RecNheI_HPV16-A1E7 Forward; RecBamHI_HPV16-A1E7 Reverse (Table S1). The HPV16 E7 PCR product was cloned into the NheI and BamHI sites of the pcDNA3.1(+) IRES GFP vector (Addgene, Watertown, MA, USA) using the CloneEZ^®^ PCR Cloning Kit according to the manufacturer’s instructions (GenScript, Piscataway, NJ, USA). E7 protein expression was confirmed by Western blotting, with three positive control vectors from Addgene, CMV16 E7 (WT), CMV16 E7 C91S (MT1), CMV16 E7 del DLYC (MT2) in 293T (Table S2). In addition, we tested three commercial antibodies to detect HPV16 E7 expression 48 h after transfection (Table S2). We found that the antiHPV16 E7 (716-325) antibody (Santa Cruz, Dallas, TX, USA) is more sensitive and specific for proteins extracted from the transfected 293T and cervical cancer cell lines, CaSki (HPV16), SiHa (HPV16), and HeLa (HPV18).

### 2.2. Mutant Construction

The pcDNA3.1(+) IRES GFP-HPV16 E7 WT was used as a template to generate variant constructs using the CloneEZ^®^ PCR Cloning (GenScript, Piscataway, NJ, USA) and site-directed mutagenesis methods (Agilent, Santa Clara, CA, USA) according to the manufacturer’s instructions. The oligonucleotides used to generate the mutations are listed in Table S1. All resulting constructs were sequenced. The empty vector and wild-type E7 constructs were used as negative and positive controls.

### 2.3. Cell Culture and Transfection

The cancer cell lines CaSki (RRID: CVCL_1100), SiHa (RRID: CVCL_0032), HeLa (RRID: CVCL_0030), and HEK293T (RRID: CVCL_0063), as well as mouse fibroblast NIH-3T3 (RRID: CVCL_0594) cells, were obtained from ATCC and cultured according to the provider’s instructions and were regularly tested for mycoplasma infection. The identify of CaSki, SiHa, and NIH-3T3 cells have been validated by Identifiler (ThermoFisher, Waltham, MA, USA) typing in the last three years. To determine HPV16 E7 protein expression in vitro, HeLa and 293T cells were seeded in 6 well plates at 2.5 × 10 ^5^ cells per well and transfected after 48 h with 3 µg of plasmid DNA using HilyMax (Dojindo, Kumamoto, Japan) transfection reagents according to the manufacturer’s instructions. The empty vector and wild-type E7 constructs were used as negative and positive controls, and the transfection efficiency of the mutants was assessed by imaging green fluorescent protein (GFP) signals through fluorescent microscopy with a ZOE Fluorescent Cell Imager (BioRad, Hercules, CA, USA) (Supplementary Materials Figure S2C). At 48 h after transfection, cells were harvested, and nuclear and cytoplasmic fractions were separated using the CelLyticTM NuCLEAR Extraction Kit (Sigma-Aldrich, St. Louis, MO, USA). Protein concentrations were determined with the Bio-Rad protein assay using the standard curve method (Bio-Rad Laboratories, Hercules, CA, USA) [21,22]. Transfection efficiency was estimated by examining green fluorescence from the GFP gene on the vector on ZOE-Fluorescent Cell Imager (Bio-Rad).

### 2.4. Western Blotting

Nuclear and cytoplasmic extracts were prepared from transfected HeLa, 293T, and NIH 3T3 cell lines using the CellLytic NuCLEAR extraction kit (Sigma-Aldrich). Protein concentration was measured with a Bio-Rad protein assay. The nuclear and cytoplasmic lysates were resolved on 10% NuPAGE Novex Bis-Tris polyacrylamide gels (Invitrogen, Waltham, MA, USA), transferred to a PVDF membrane, blocked at room temperature for two hours in 5% milk in Tris-buffered saline with 0.1% Tween 20 (TBST), and then blotted. Western blot analysis was performed with the following antibodies: HPV16 E7 (716-325) mouse monoclonal antibody at 1:100 (Santa Cruz). In addition, we selected rabbit monoclonal antibody at 1:1000 of TBP (D5G7Y) and beta-Tubulin (9F3) (Cell Signaling, Danvers, MA, USA) as loading controls for nuclear and cytoplasmic proteins for WB, respectively. Densitometric analysis of specific signals using Image Lab software 6 on three independent blots was quantitated and normalized to the loading control TBP and beta-Tubulin, respectively. The nuclear and cytoplasmic proteins of HPV16 E7 wild type were considered as 100% expression. Original Western blot images are found in Supplementary Figure S1.

### 2.5. Determination of Morphological Transformation

NIH3T3 cells stably transfected with the empty vector, wild type E7, and the indicated E7 variants were plated in 75 mL flasks at an equal cell density in DMEM medium with G418 500µg/mL. At 60% confluence, cell morphology was examined and imaged under a microscope with 10× and 20× magnification (Zeiss Axio, Carl Zeiss, Oberkochen, Germany). NIH3T3 cells were used in an HPV16 transformation assay by examining anchorage-independent growth in soft agar. NIH3T3 cells were transfected with pcDNA3.1(+) IRES GFP-HPV16 E7 vectors as described above. At 48 h after transfection, cells were selected in a medium containing G418. After three weeks of selection, the stably transfected cells were seeded into soft agar in 96-well plates.

### 2.6. Anchorage-Independent Cell Growth

We used the Cytoselect 96-well Cell Transformation Assay (Cell Biolabs, San Diego, CA, USA) to assess colony formation according to the manufacturer’s instructions. We counted live cells (variability >95%) using the Countess automated cell counter (Invitrogen, Waltham, MA), and each cell line was counted twice. The cells were seeded in soft agar at a density of 1 × 10 ^4^ cells per well, and duplicate plates were tested using Quantitation of Anchorage-Independent Growth and colony formation. The colony formation was quantified by solubilizing the soft agar, lysing the cells, and incubating cell lysates with the CyQUANT GR Dye (Cell Biolabs) with the GLOMAX Multi Detection System (Promega, Madison, WI, USA). In addition, the colonies were imaged under a microscope (Zeiss Axio, Carl Zeiss, Oberkochen, Germany).

### 2.7. Wound Healing Assay

The migration of NIH3T3 stably transfected cells was monitored by the Lionheart FX Automated Live Cell Imager (BioTek, Winooski, VT, USA), and cellular analysis of the kinetic images was performed using Gen5 software. Cells were grown in T75 flasks until they reached 60–75% confluency. The cells were detached from the flask using phenol red-free TrypLE Express Trypsin (Gibco/Thermo Fisher). The method for cell counting was the same as described above, and cells were seeded in 24-well plates with 1.0 × 10^5^ live cells per well. Cell monolayers were scratched using an Auto-scratch machine (BioTek). The plate was placed in the Lionheart FX Automated Live Cell Imager in 5% CO_2_ at 37 °C. The wound area was imaged every 2 h for 20 h using a 4× objective and phase contrast, and bright field illumination. The number of cells migrating into the wound area was counted using percent confluency within the wound area (www.biotek.com (accessed on 4 October 2022)).

### 2.8. Statistical Analysis

Mann–Whitney-U tests and unpaired two-tailed *t*-tests were performed using GraphPad Prism version 8 for Windows 10; *p*-values of <0.05 were considered significant.

## 3. Results

### 3.1. HPV16 E7 Mutagenesis and Protein Expression

To test E7 protein expression and function of 18 specific E7 variants, we generated expression constructs in the pcDNA3.1(+) IRES GFP vector. We constructed nine variants using the ClonEZ PCR cloning method, nine by site-directed mutagenesis, and all clones were confirmed by DNA sequencing. Figure 1 shows the distribution of variants and the E7 protein functional domains. Eleven variants were in CR3, and three and four in CR1 and CR2, respectively. A summary of all the tested E7 mutants and their cancer association and protein expression are shown in Table 1. The HPV16 E7 level of variants found in precancers is significantly higher than in the control (cancer-free) group (Table 1).

To determine if the variants affect the level of steady-state E7 protein, we transiently transfected them into HeLa, and 293T cells and nuclear and cytoplasmic proteins were detected by Western blotting (WB). These cell lines were chosen because HeLa cells are derived from cervical cancer and have an intact pRB tumor suppressor pathway [23,24,25,26], and 293T is an HPV-negative cell line derived from a human fetus and has high transfectability.

The anti-HPV16 E7 (716-325) antibody was tested for sensitivity and specificity with 293T transfected with three positive control vectors and in cervical cancer cell lines containing HPV16 (CaSki and SiHa) or HPV18 (HeLa) (Figure S1 and Table S2). The positive controls and our WT E7 vector displayed a correctly sized E7 protein (Figure S1A). Similarly, the HPV16 E7 antibody detected the endogenous HPV16 E7 protein in the cervical cancer cell lines CaSki (600 copies of HPV16) and SiHa (1–2 copies of HPV16), but not the HPV18 E7 protein in HeLa cells (Figure S1B). Therefore, the anti-HPV16 E7 (716-325) antibody is sensitive and specific for HPV16 E7 protein, consistent with published data [27,28].

### 3.2. Reduced Protein Expression Levels of HPV16 E7 Variants

**Figure 2:**
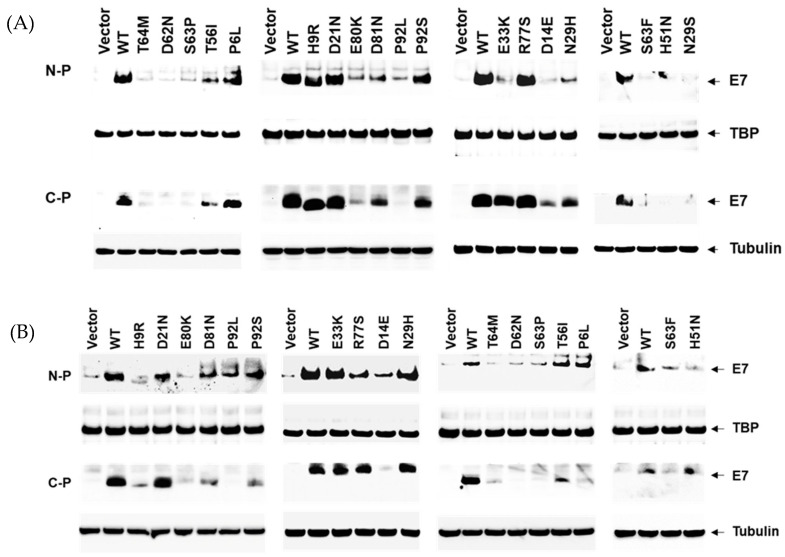
Western blot analyses of HPV16 E7 from HeLa (**A**) and 293T (**B**) cells transiently transfected with empty vector, wild-type and indicated mutant E7 constructs. The position of the E7, Tubulin and TBP (TATA binding protein) are indicated. N-P and C-P represent the nuclear and cytoplasm proteins, respectively. Statistical analysis of three independent experiments is shown in Figure S2.

The HPV16 E7 protein has been previously localized to the cytoplasm and nucleus and observed as a dimer in the nucleus [29,30,31,32,33]. To determine the location of our HPV16 E7 WT and mutant proteins, we separated cytoplasmic and nuclear fractions and observed the E7 protein in both fractions in HeLa and 293T cells (Figure 2). For the 13 E7 variants previously identified in controls [34], seven (54%), N29S, E33K, D62N, S63F, S63P, T64M, and P92L, showed deficient levels of steady-state E7 protein in the nucleus as compared with E7 WT protein, and the four remaining (36%), T56I, E80K, D81N, and P92S, had moderately reduced levels (Figure 2A and Figure S2A). In 293T cells, 9 out of 10 (90%) of the variants tested in E7 identified in controls also showed decreased E7 protein levels as compared to WT in both nuclear and cytoplasmic fractions (Figure 2B and Figure S2B). The transfection efficiency or the WT and mutant E7 vectors was determined by counting GFP-positive cells, using the GFP expressed derived from the same vector (Supplementary Figure S2C).

Similarly, we found a severe to moderate reduction of E7 protein levels for the D14E, N29H variants identified in CIN2 and H9R and E33K in controls, in either HeLa or 293T cells (Figure 2A,B and Figure S2A,B). Two of the three precancer variants tested, P6L and R77S, showed E7 levels similar to WT E7, and H51N showed moderately reduced levels (Figure 2A, Table 1). P6L is adjacent to another mutation (T5K) with transforming potential like E7 WT [13].

### 3.3. HPV16 E7 Variants Not Associated with Cancer Have a More Normal Cell Morphology and Reduced Anchorage-Independent Growth in NIH3T3 Cells

**Figure 3:**
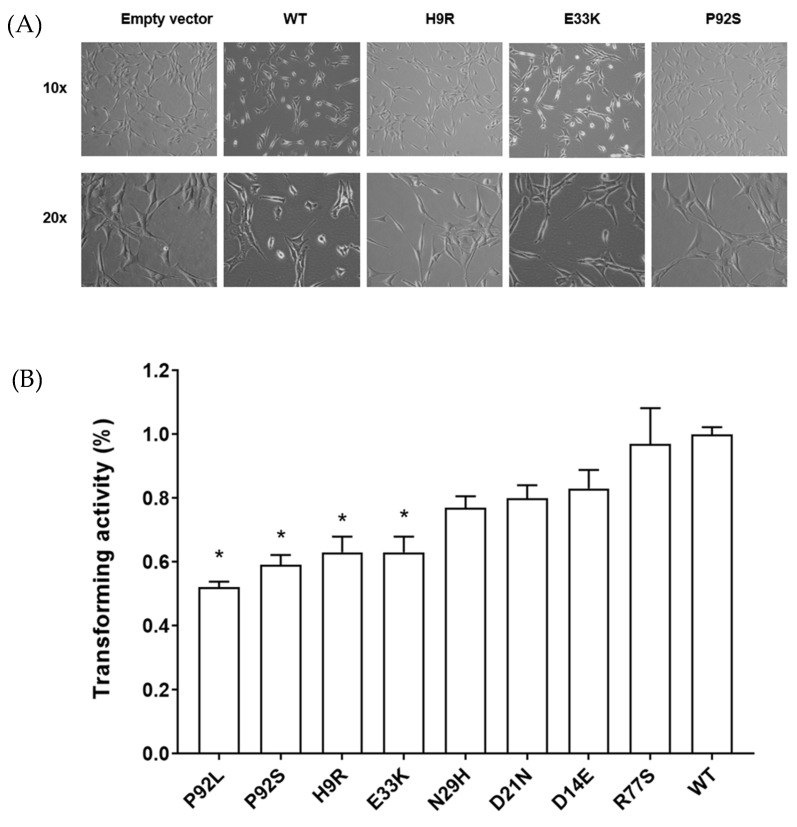
Morphology and anchorage-independent growth of NIH3T3 transfected with HPV16 E7 WT and variant types. (**A**). Morphology of NIH3T3 cells stably expressing empty vector, WT and the indicated variant types. Morphology of stable NIH3T3 cells (10x, 20x). (**B**). Quantitation of anchorage-independent cell growth of HPV16 E7 variants on soft agar and each variant was compared to WT that cell growth was adjusted to 100%. *, *p* < 0.05 for comparison with WT cells.

To test the ability of HPV16 E7 transfected cells in a cellular transformation assay, we chose a representative subset of variants, five from the control group [H9R, D21N, E33K, P92L, P92S] and three from the CIN2 or CIN3 precancer groups [D14E, N29H, R77S], to assess cell morphology and growth in NIH3T3 cells, an established model of transforming gene function. As shown in Figure 3A, E7 WT cells displayed a spindle-shaped morphology and more rounded, mitotic cells. However, the control variant (H9R, P92S) cells and empty vector-transfected cells had a near-normal morphology, and E33K cells displayed an intermediate phenotype.

We assessed growth and colony size in soft agar to determine the effects of HPV16 E7 variants on cellular transformation. NIH3T3 cells or empty vector-transfected cells display few colonies of small size, whereas WT E7-transfected cells showed an increased number of colonies and of a larger size (Figure S3). We used a soft agar growth assay, quantitated with a fluorescent dye, to assess abnormal cell growth. Figure 3B shows the quantification of the colony size, including a WT E7 positive control and a mutation in the CXXC domain known to be non-functional as a negative control (C in CXXC) from reference [35]. All four variants with significantly reduced (50–60%) transforming activity (H9R, E33K, P92L, and P92S) come from control subjects. Two of the three variants with 70–80% transforming activity are from CIN2 subjects and the R77S variant, from a CIN3 precancer subject, has similar activity as the WT (Figure 3B). Therefore, 80% of the control mutants tested caused significantly reduced anchorage-independent growth activity.

### 3.4. HPV16 E7 Variants Display Decreased NIH3T3 Cell Migration

**Figure 4:**
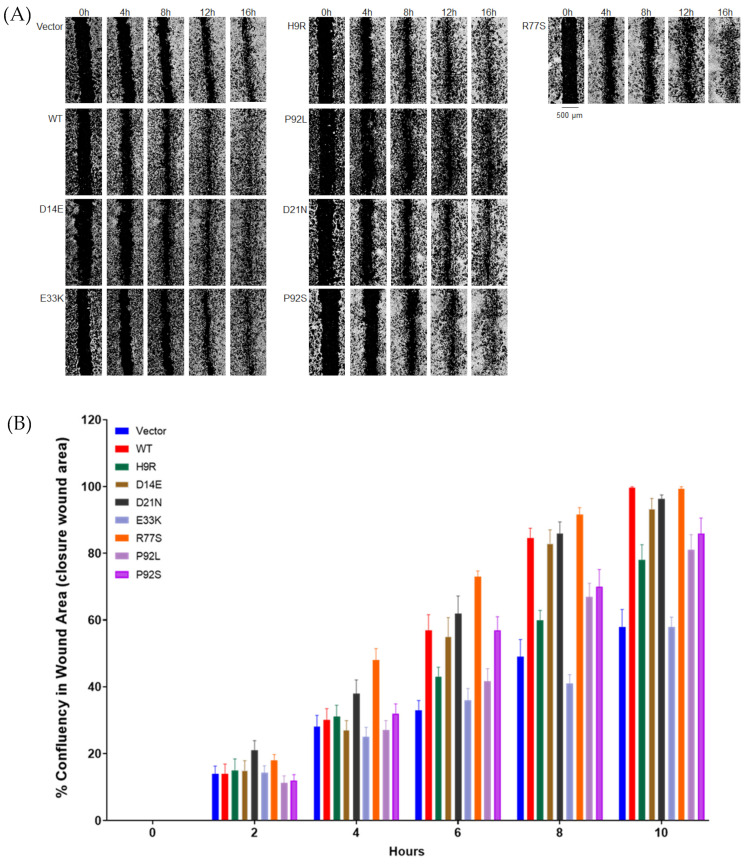
Migration of stably transfected NIH3T3 cell lines in a wound healing (scratch) assay. Stable NIH3T3 cell clones were plated onto 24-well plates. Wounds in the monolayer were created with an automated tool, plates were washed to remove detached cells, and closure of the wound was followed for 24 h with hourly imaging in a BioTek Lionheart FX Automated Live Cell Imager. Images are shown in (**A**) and quantitation over time in (**B**). Results are expressed relative to WT E7 migration at 10 h. At 10 h: WT vs. Vector (*p* = 0.0022); WT-H9 R (*p* = 0.0079); WT-E33K (*p* = 0.0022); WT-D14E (*p* > 0.05); WT-P92L (*p* = 0.0080) WT-P92S (*p* = 0.0082). If we apply a highly conservative Bonferroni multiple testing correction of six tests of mutant versus WT, only vector and E33K remain significant.

To determine the effect of a representative subset of seven variants (those noted above, excluding N29H) on cell migration in a wound-healing assay, we performed automated scratches of a cell monolayer, monitoring cell migration every two hours over 20 h. HPV16 E7 WT and R77S (from CIN3 precancer) NIH3T3 cells showed increased migration into the wound area as compared with H9R (CIN2), E33K (CIN2), P92L (Control), and P92S (Control) and moderately increased compared with D21N (Control) and D14E (CIN2; Figure 4). The percentage of cell confluency in the wound area was calculated from images taken every 2 h in a LionHeart FX Automated Live Cell Imager in triplicate. Analysis of the time course of wound closure showed that WT and R77S (CIN3) cells displayed significantly increased migration compared to H9R, E33K, P92L, and P92S, from controls. In addition, H9R, P92L, and P92S showed somewhat faster wound closure than empty vector-transfected cells, and in this assay, the E33K variant was indistinguishable from the empty vector. These results confirm that four of the six HPV16 E7 control or CIN2 group mutants have decreased activity of the E7 protein, as measured by NIH3T3 cell migration (Figure 4B). The data from all the tested E7 mutants, associated phenotypes, and assay results are summarized in Table 2. Using extended follow-up data from these individuals (see [20]), we found that 5/13 (38%) of the subjects from the control group were HPV negative on a subsequent visit and therefore cleared their HPV16 infection (Table 2).

## 4. Discussion

The E7 oncogene is one of the two most important genes contributing to the cellular transformation and oncogenicity of high-risk HPV types. E7 is essential for the continued oncogenic growth of cervical cancer cells [37]. As HPV16 is the most carcinogenic HPV type, accounting for over half of all cervical cancer cases worldwide, it is critical to understand the activities of this protein. There are now multiple reports demonstrating a lack of genetic variation in the E7 protein in HPV16 isolates from cervical precancer and cancer, as well as head and neck cancer [20,36]. The most extensive study of 5570 HPV16 genomes demonstrated a 6 to 7-fold increase in HPV16 E7 variants in isolates from women without cancer [20]. Our data presented here provides a partial mechanistic explanation for this observation.

The study aimed to assess the role of E7 amino acid variants previously identified [20] in HPV-infected, cancer-free controls and precancer cases to understand how these alterations may affect cervical carcinogenesis. The approaches used include transfecting HeLa, 293T, and NIH3T3 cells with expression vectors containing either WT or mutant forms of E7. We tested these variants for alteration in the steady-state levels of E7 protein in the cytoplasm and nucleus, as well as their efficiency to alter morphology, anchorage-independent growth, and migration in stably transfected NIH3T3 cell clones. We found that most of the E7 variants identified in controls have lower steady-state protein levels, in both the cytoplasm and the nucleus, in a cervical cancer cell line (HeLa) and non-cervical cells (293T). We have not determined whether the lower protein levels are due to alterations in translation, stability, or protein degradation. Still, the reduced levels, especially in the nucleus, are consistent with a reduced transforming ability.

The level of E7 protein does not always reflect the transforming activity of the protein, and we, therefore, used multiple methods to assess E7 activity (data summarized in Table 2). Anchorage-independent growth is an informative in vitro assay to detect transformation activity [38]. Several mutants found exclusively in controls (H9R, E33K, P92L, and P92S) show 30–50% lower transforming activity, whereas the D14E and R77S mutants found in precancers have 80–100% activity in this assay. E33K is in the CR2 domain and is immediately downstream of the two known CKII phospho-acceptor sites at S31 and S32 [39]. Therefore, its reduced transforming activity could be due to an alteration in phosphorylation. However, we also found that the E33K mutant appears to be defective in nuclear localization in HeLa cells. It is in a region known to be critical for E7 localization to the nucleus (aa 1–37) [40]. The P92L and P92S mutants affect a conserved proline inside the C-terminal CXXC motif and may cause subtle alterations in E7 structure or stability [41].

The D14E mutant, seen in a single CIN2 subject, has a low steady-state protein level but a modestly reduced activity (~80%) in the soft agar assay. Aspartic and glutamic acid are similarly charged amino acids, and this alteration may only slightly affect E7 activity. Likewise, the other alterations identified in CIN2 or CIN3 precancer (P6L, N29H, H51N, and R77S) appear to be residues that can tolerate change. Gillison et al. [36] identified an H51N containing HPV16 isolate in a head and neck tumor, consistent with this variant being compatible with cancer.

We also assessed the activity of stably transformed NIH3T3 cells to migrate in culture using the wound-healing (scratch) assay. Using an instrument that automatically generates uniform scratches and continuously monitors cell growth over 20 h we show a dramatic difference between NIH3T3 cells containing an empty vector and those with the wild-type E7 construct. Scratch assays have been used to monitor oncoprotein E6 and E7 function and demonstrate that HPV16 oncoproteins promote cell migration and invasion [41,42]. Still, to our knowledge, this is the first study to employ the assay to study E7 mutants in NIH3T3 cells. The wound-healing activity of the E7 mutants gave very similar results as the anchorage-independent growth assay, with R77S showing activity equal (or perhaps stronger) than wild type; D14E and N29H are slightly impaired, whereas E33K displayed migration similar to the empty vector control.

Interestingly, the previous finding that an alteration of the highly conserved arginine at residue 77 to glutamic acid (R77E) [43] has a high steady-state protein level and promotes migration equal to or better than wild type. Furthermore, R77 is in an alpha-helical domain and does not directly touch pRB or E2F1, and R77E increases E7 pRB binding capacity [16]. Therefore, the effect of the R77S variant in our study on pRB binding would be interesting to explore further.

Notably, no variants in functional or structural E7 motifs, including the conserved residues of the LxCxE and CXXC motifs, have been found in patients. Mutations in these residues, C24G, E26G for LxCxE, and C58G and C91G for CXXC, dramatically reduce or eliminate E7 transforming activity [35,44,45]. E7 is a zinc-binding protein, and the cysteine residues in the zinc-finger binding domain (C58, C59, C61, C91, C94) are essential for E7 function. Therefore, it is likely that the variants identified in actively infected, cancer-free patients, do not involve amino acid residues of E7 that are essential for function. However, many of the variants found in controls are in amino acids in CR3 (P92L/S) or near the CXXC motifs (T56I, D62N, S63P/F, T64M) (Figure 1). This result suggests that alterations of residues near the CXXC motifs alter the structure of E7 and partially reduce function. Other variants near the LxCxE and CKII motifs partially reduce cellular oncogenicity [35,44,45]. It is also possible that some E7 variants produced peptides recognized by T cells, leading to viral clearance. The precise molecular mechanism of action of variants of E7 in cervical cancer development needs to be further explored [16,41,42].

The limitations of this study are that we did not determine the molecular basis for the reduced steady-state level of protein and performed functional analyses with stable clones on a subset of the variants. In addition, our functional assays involve NIH3T3 mouse fibroblast cells, and it would be informative to repeat these results using viral constructs in primary cells or an infectious model of HPV [35,44,45]. However, the data are consistent with the paradigm that many amino acid-altering E7 variants in controls have attenuated migration and transforming ability.

## 5. Conclusions

In conclusion, cervical cancer development appears to require the continuous activity of a fully functional E7 protein. Our genetic epidemiologic data previously demonstrated that variation in E7 is poorly tolerated [21]. Our results suggest that even a 50% inhibition of E7 activity would render HPV defective for oncogenesis. These alterations may generate an HPV16 protein similar to low-risk HPV E7 proteins that are less efficient in binding and degrading pRB [13,46]. Our results suggest that inhibitors of HPV16 E7 expression, stability, or activity could play a role in cervical cancer prevention or treatment.

## Figures and Tables

**Table 1:** HPV16 E7 variants tested and associated nuclear protein levels of E7.

Variants	Original Codon	Mutant Codon	Nucleotide Change	Control (No.)	Precancer (No.)	E7 *
Wild type						1
**Control**						
H9R	CAT	CGT	A > G	1	0	0.8
D14E	GAT	GAA	T > A	0	1 (CIN2 **)	0.11
D21N	GAT	AAT	G > A	1	0	0.92
N29H	AAT	CAT	A > C	0	1 (CIN2 **)	0.3
N29S	AAT	AGT	A > G	2	0	0.018
E33K	GAG	AAG	G > A	2	0	0.2
T56I	ACC	ATC	C > T	1	0	0.55
D62N	GAC	AAC	G > A	1	0	0.11
S63F	TCT	TTT	C > T	1	0	0.1
S63P	TCT	CCT	T > C	1	0	0.3
T64M	ACG	ATG	C > T	1	0	0.11
E80K	GAA	AAA	G > A	1	0	0.26
D81N	GAC	AAC	G > A	1	0	0.42
P92L	CCC	CTC	C > T	2	0	0.1
P92S	CCC	TCC	C > T	2	0	0.68
**Precancer**						
P6L	CCT	CTT	C > T	1	1 (CIN3 **)	1.1
H51N	CAT	AAT	C > A	6	3 (CIN3/AIS ***)	0.46
R77S	CGT	AGT	C > A	1	1 (AIS)	0.99

*: *p* < 0.05 (E7 control ratio vs. E7 precancer ratio). (Ratio from E7 variants/E7 wild types in the control and precancer groups, respectively). **: Cervical intraepithelial neoplasia (CIN)\2 or 3; ***: AIS, Adenocarcinoma in situ. Columns 2–4 information from reference [20].

**Table 2:** Summary of the HPV16 E7 variants, protein expression and functional assays.

Construct	Cervical Disease	HPV16 Clearance	Function Domain	Nuclear Protein	Transforming Activity (% Growth in Soft Agar)	Wound Healing
Vector						+
D81N	Control		CR3	++	NA	NA
E80K	Control		CR3	++	NA	NA
P92S	Control		CR3	++	++	++
S63F	Control		CR3	+	NA	NA
T56I	Control		CR3	++	NA	NA
T64M	Control		CR3	+	NA	NA
D21N	Control	yes	CR2	+++	+++	+++
D62N	Control		CR3	+	NA	NA
P92L	Control	yes	CR3	+	++	++
S63P	Control	yes	CR3	+	NA	NA
E33K	Control	yes	CR2	+	++	+
H9R	Control		CR1	+++	++	++
N29S	Control, CIN2	yes (both)	CR2	+	NA	NA
N29H	CIN2		CR2	++	+++	NA
D14E	CIN2		CR1	++	+++	+++
H51N *	CIN3/AIS		CR3	++	NA	NA
P6L	CIN3		CR1	++++	NA	NA
R77S	AIS		CR3	++++	++++	++++
**Wild type**				**++++**	**++++**	**++++**

CIN2, cervical intraepithelial neoplasia grade 2; CIN3, cervical intraepithelial neoplasia grade 3; AIS, adenocarcinoma in situ. *, also in HNSCC [36]. Green highlighting indicates a significant decrease in activity. NA, not assessed. + to ++++, represent the protein level from weak to strong.

## Data Availability

Not applicable.

## Supplementary Materials

The following are available online at https://www.mdpi.com/article/10.3390/cancers14194895/s1, Figure S1: Detection of HPV16 E7 expression level in transfected 293T cells and cervical cancer cell lines using antiHPV16 E7 (716–325) antibody. Figure S2: Quantitation of Western blot analyses of HPV16 E7 from HeLa cells transfected with empty vector, wild-type and mutant E7 constructs. Figure S3: Anchorage independent cell growth of HPV16 E7 variant stable clones grown in soft agar. Table S1: Oligonucleotide primers. Table S2: HPV16 E7 pcDNA clones and antibodies tested.
