# Supplementary material for: HPV16 E7 Nucleotide Variants Found in Cancer-Free Subjects Affect E7 Protein Expression and Transformation
Source: Cancers (Basel). 2022 Oct 6;14(19):4895. doi: 10.3390/cancers14194895 (PMC9562847; doi:10.3390/cancers14194895)
Supplement: Supplementary file 1 [file cancers-14-04895-s001.zip › cancers-1776800-supplementary.pdf]

A.

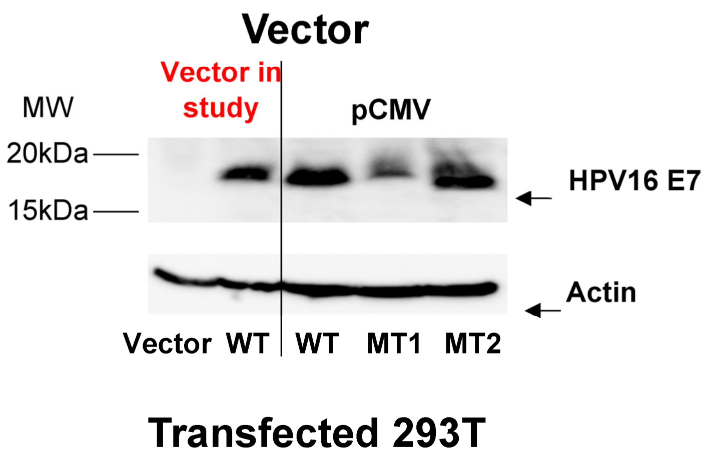
Vector
Vector in study
pCMV
MW
20kDa
15kDa
HPV16 E7
Actin
Vector WT
WT MT1 MT2
Transfected 293T

B.

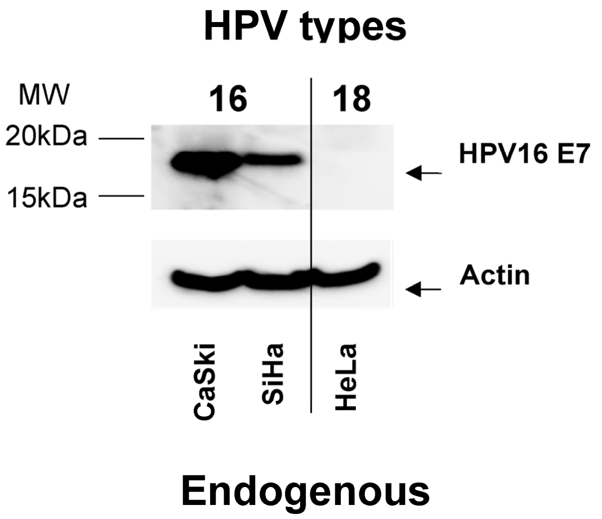
HPV types
MW
16
18
20kDa
15kDa
HPV16 E7
Actin
CaSki
SiHa
HeLa
Endogenous

C. Western Blotting original images

Test HPV16 E7 antibody raw data

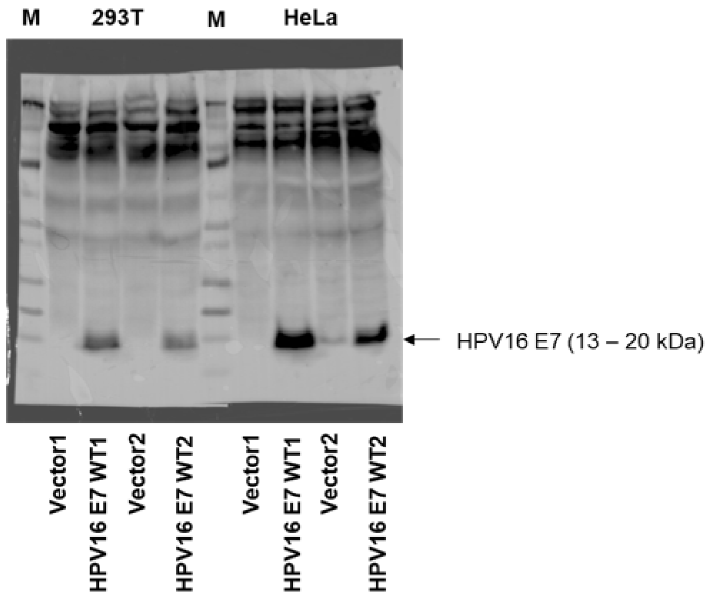

Loading amount of protein in No.1 is double than No.2

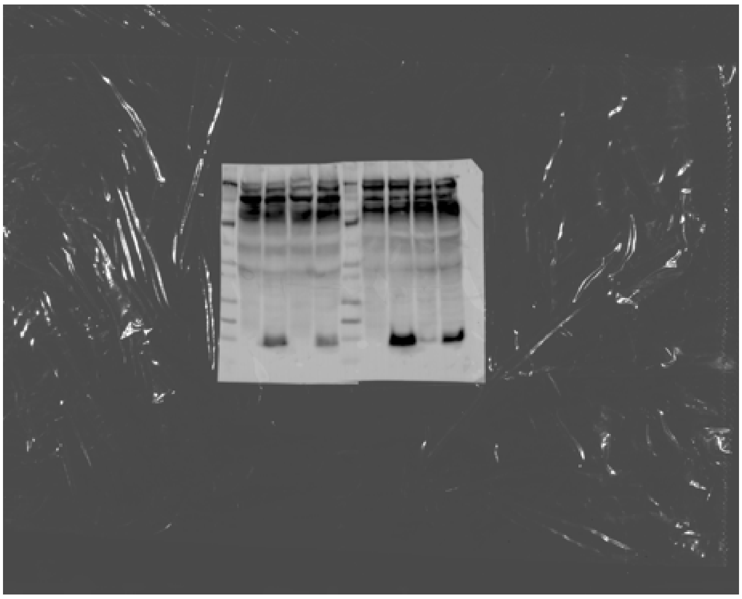

HPV16 E7 Antibody (716-325): sc-51951.

Western blot analysis of HPV 16 E7 expression

D.

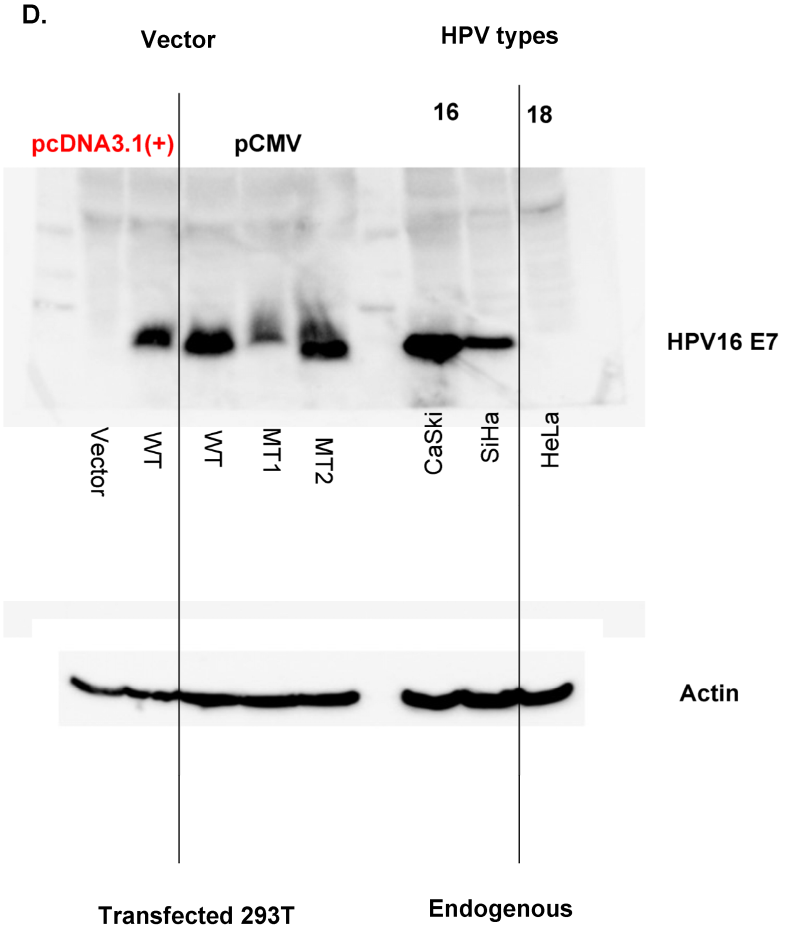

E. HeLa Western blot Raw Data

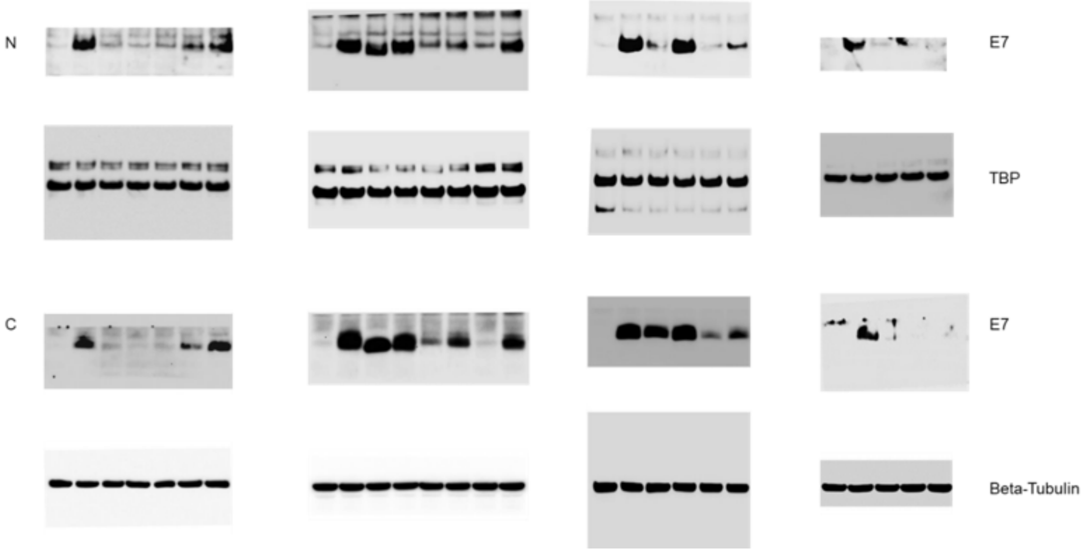

F. 293T Western blot Raw Data

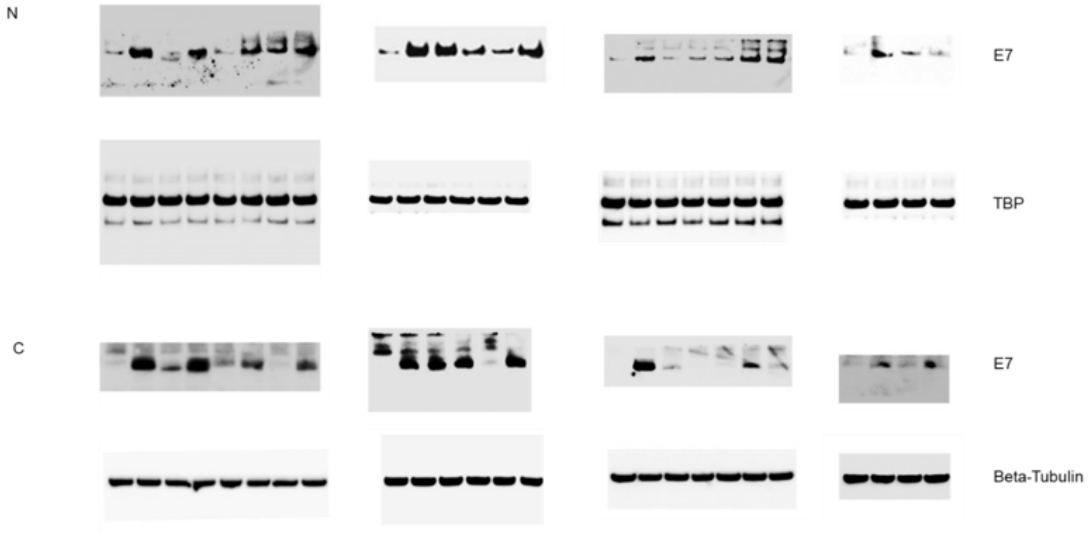

Figure S1. Detection of HPV16 E7 protein expression in transfected 293T cells and cervical cancer cell lines using the optimum antiHPV16 E7 (716-325) antibody after screening three different antiHPV16 E7 antibodies (Table S2). pcDNA3.1(+)IRES GFP-HPV16 E7 WT and positive control, pCMV-HPV16 E7 WT, MT1 (C91S) and MT2 (del DLYC) expressed in transient transfected 293T cells after 48h in culture (A). Endogenous HPV16 E7 proteins were detected in HPV16 cervical cancer cell lines only, CaSki (600 copies/per cell), SiHa (1-2 copies/per cell), not detected in the HPV18 cervical cancer cell line, HeLa (B). The results indicated that the antiHPV16 E7 (716-325) antibody has high sensitivity and specificity. (C) Western Blotting original images. Test HPV16 E7 antibody raw data. (D–F) Western blot original images. Blots were cut into strips to probe with multiple antibodies to proteins of differing size to avoid stripping and reprobing.

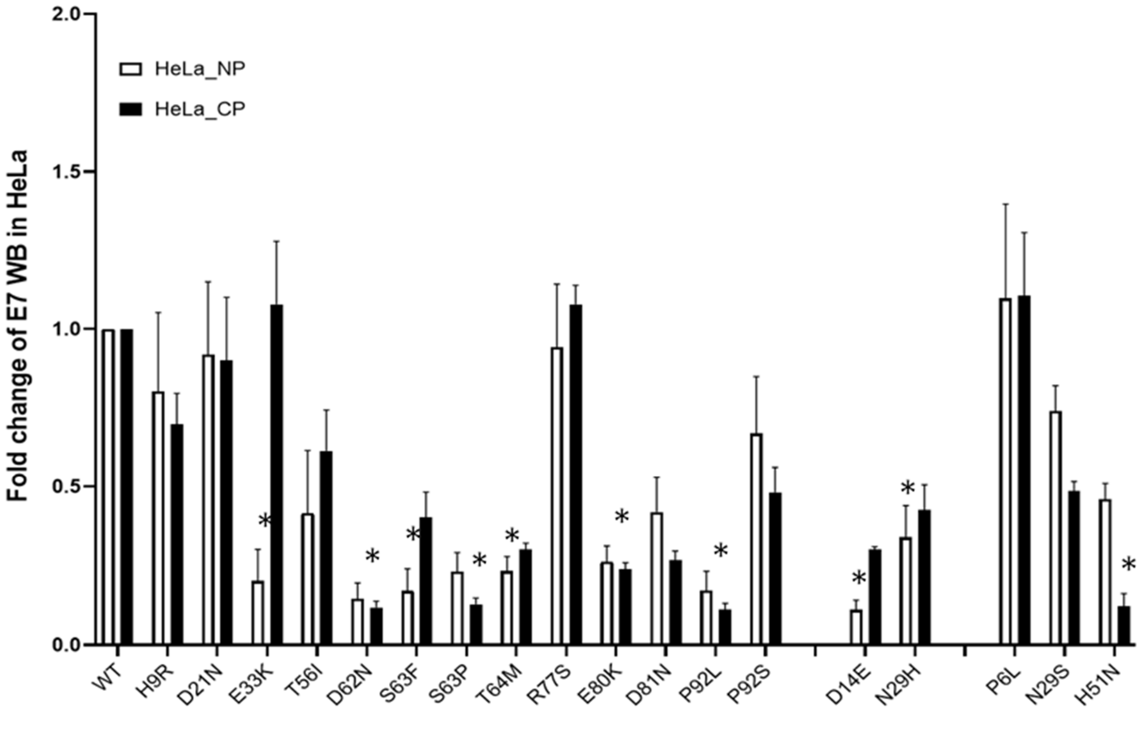

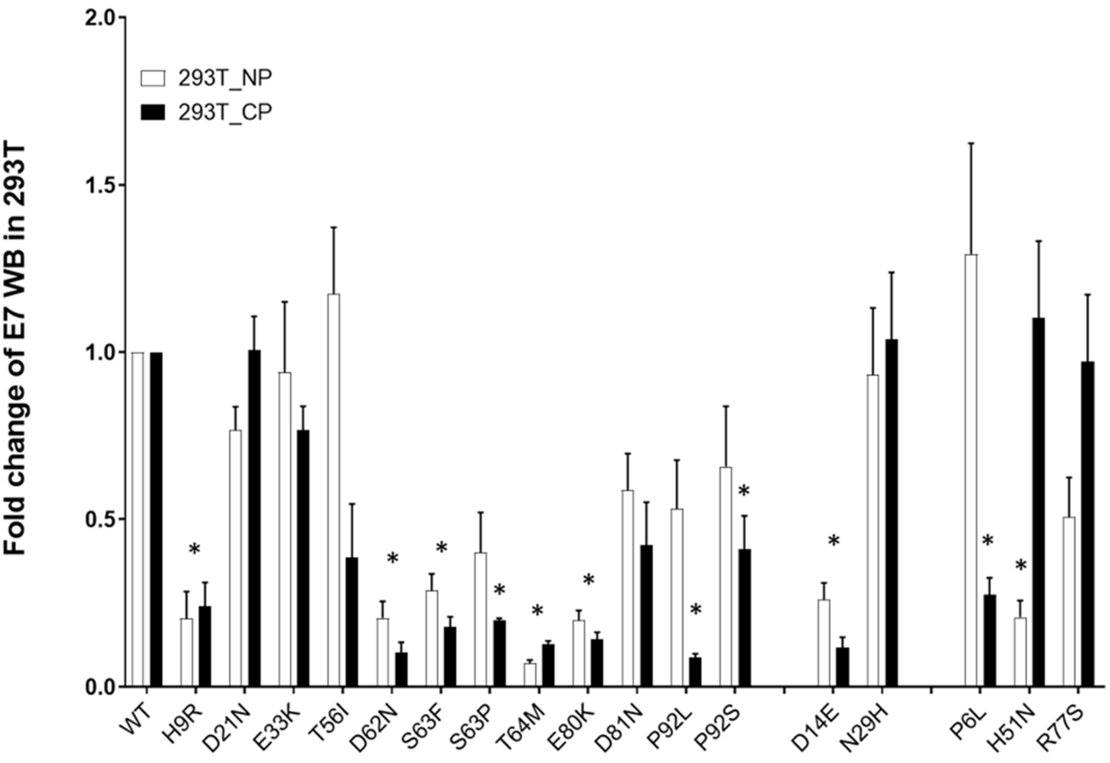

Figure S2. Quantitation of western blot analyses of HPV16 E7 variants from HeLa (A) and 293T (B) cells transiently transfected with empty vector, wild-type and mutant E7 constructs. *: represents significantly lower E7 protein levels as compare with to wild type E7 (p<0.05). The results represent the mean of three independent experiments.

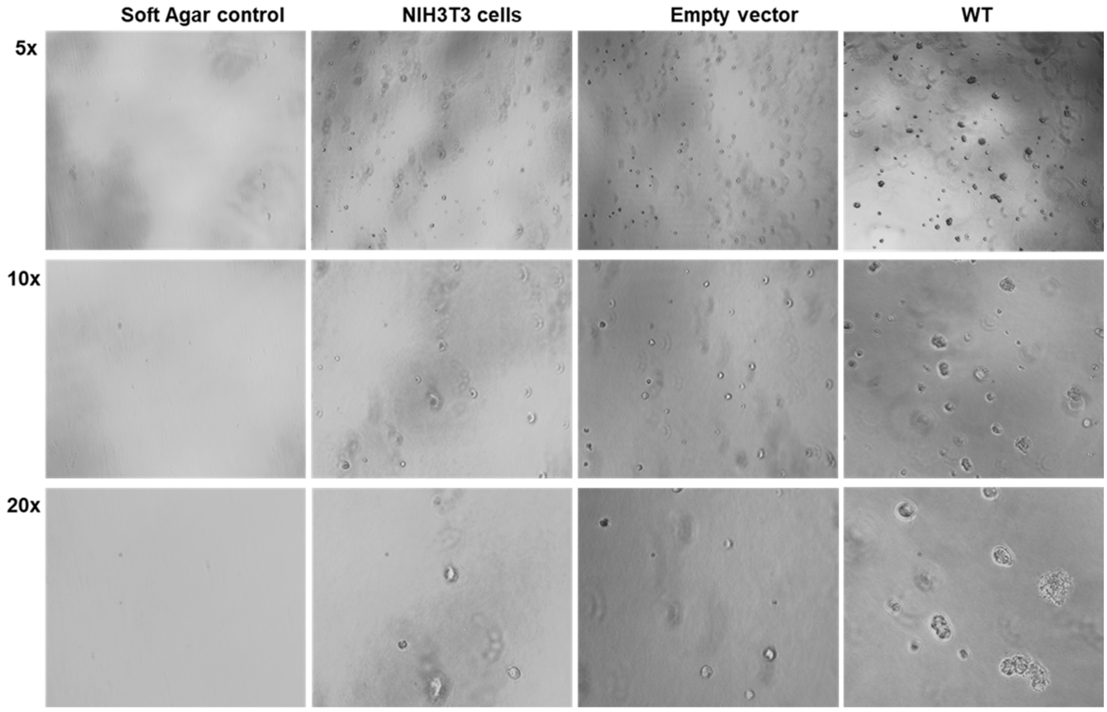

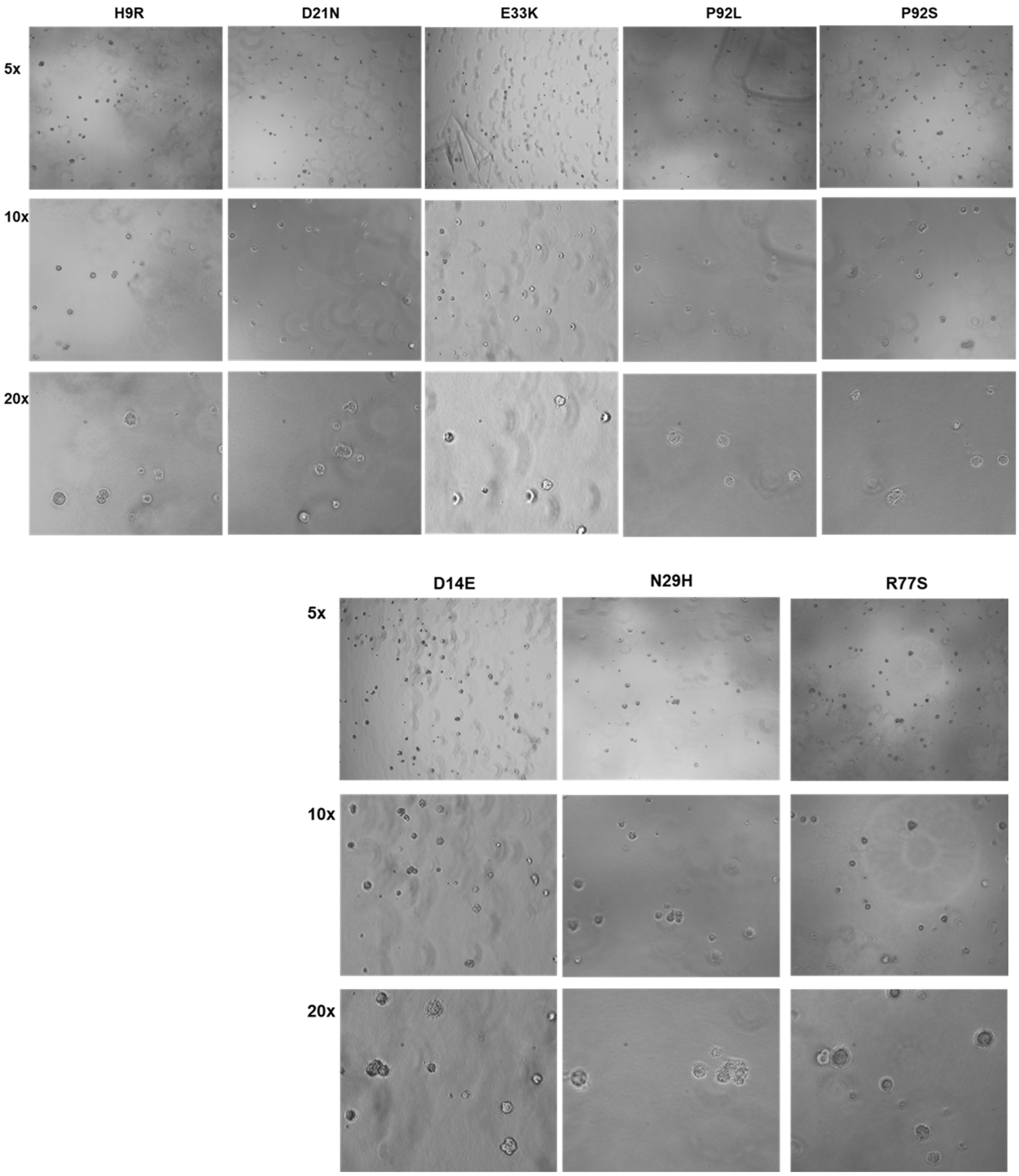

Figure S3. Anchorage independent cell growth of HPV16 E7 variant stable clones grown in soft agar. NIH3T3 cells stably transfected with the empty vector, WT and the indicated variants. Colonies formed after 20 days of culture were imaged with 5x, 10x, 20x magnification.

Table S1. Oligonucleotide primers.

Oligo Name	Sequence (5' to 3')
**Oligonucleotides used for pcDNA3.1+IRESGFP_HPV16E7**	
RecBamHI_HPV16-A1E7_Rev.	5'-CCACACTGGACTAGT GGATCC TTATGGTTTCTGAGAACAGATGGGGCA-3'
RecNheI_HPV16-A1E7_For	5'-GGGAGACCCAAGCTG GCTAGC GCCACCATGCATGGAGATACACCTACATTG-3'
**Oligonucleotides used for CloneEZ® PCR Cloning**	
HPV16E7P6LFor	5'-GGGAGACCCAAGCTGGCTAGCGCCACCATGCATGGAGATACACTTACATTGCATGAATATATG-3'
HPV16E7H9RFor	5'-GGGAGACCCAAGCTGGCTAGCGCCACCATGCATGGAGATACACCTACATTGCGTGAATATATG-3'
HPV16E7D14EFor	5'-GGGAGACCCAAGCTGGCTAGC*GCCACC* ATGCATGGAGATACACCTACATTGCATGAATATATGTTAGAATTGCAACCAGAG-3'
HPV16E7D21NFor	5'-GGGAGACCCAAGCTGGCTAGC*GCCACC* ATGCATGGAGATACACCTACATTGCATGAATATATGTTAGATTTGCAACCAGAGACAACTAATCTCTACTG-3'
HPV16E7P92SRev	5'-CCACACTGGACTAGTGGATCCTTATGGTTTCTGAGAACAGATGGAGCACACAATTCC-3'
HPV16E7P92LRev	5'-CCACACTGGACTAGTGGATCCTTATGGTTTCTGAGAACAGATGAGGCACACAATTCC-3'
HPV16E7D81NRev	5'-CCACACTGGACTAGTGGATCCTTATGGTTTCTGAGAACAGATGGGGCACACAATTCCTAGTGTGCCCATTAACAGGTTTTCCAAAGTACG-3'
HPV16E7E80KRev	5'-CCACACTGGACTAGTGGATCCTTATGGTTTCTGAGAACAGATGGGGCACACAATTCCTAGTGTGCCCATTAACAGGTCTTTCAAAGTACG-3'
HPV16E7R77SRev	5'-CCACACTGGACTAGTGGATCCTTATGGTTTCTGAGAACAGATGGGGCACACAATTCCTAGTGTGCCCATTAACAGGTCTTCCAAAGTACTAATGTCTACG-3'
Oligonucleotides used for site-directed mutagenesis	
HPV16E7N29H_Rev	5'-ctctgagctgtcatgtaattgctcataacagtagagatcag-3'
HPV16E7N29H_For	5'-ctgatctctactgttatgagcaattacatgacagctcagag-3'
HPV16E7N29S_Rev	5'-ctctgagctgtcatctaattgctcataacagtagagatcag-3'
HPV16E7N29S_For	5'-ctgatctctactgttatgagcaattagatgacagctcagag-3
HPV16E7H51N_Rev	5'-aaggttacaatattgtaattggctctgtccggttctgc-3'
HPV16E7H51N_For	5'-gcagaaccggacagagccaattacaatattgtaacctt-3'
HPV16E7E33K_Rev	5'-ttcatcctcctccttgagctgtcatttaattgctcataaca-3'
HPV16E7E33K_For	5'-tgttatgagcaattaaatgacagctcaaaggaggaggatgaa-3'
HPV16E7T56I_Rev	5'-gagtcacacttgcaacaaaagattacaatattgtaatgggctct-3'
HPV16E7T56I_For	5'-agagcccattacaatattgtaatcttttgttgcaagtgtgactc-3'
HPV16E7D62N_Rev	5'-ccgaagcgtagagttacacttgcaacaaaaggttacaatatt-3'
HPV16E7D62N_For	5'-aatattgtaaccttttgttgcaagtgtaactctacgcttcgg-3'
HPV16E7S63F_Rev	5'-cgcacaaccgaagcgtaaagtcacacttgcaacaa-3'
HPV16E7S63F_For	5'-ttgttgcaagtgtgactttacgcttcggttgtgcg-3'
HPV16E7S63P_Rev	5'-gcacaaccgaagcgtagggtcacacttgcaacaaa-3'
HPV16E7S63P_For	5'-tttgttgcaagtgtgaccctacgcttcggttgtgc-3'
HPV16E7T64M_Rev	5'-acgcacaaccgaagcatagagtcacacttgcaac-3'
HPV16E7T64M_For	5'-gttgcaagtgtgactctatgcttcggttgtgcgt-3'

Table S2. HPV16E7 pcDNA clones and antibodies tested.

	Description of product	Catalog No.	Company
**Positive controls**	CMV 16 E7	13686	Addgene
	CMV 16 E7 C91S	13689	Addgene
	CMV 16 E7 del DLYC	13687	Addgene
**Vector for constructing full length HPV16 E7**	pcDNA3.1 (+) IRES GFP	51406	Addgene
**Testing antibodies**	HPV16 E7 (716-325)	sc-51951	Santa Cruz
	HPV16 E7 (ED17)	sc6981	Santa Cruz
	HPV16 E7 (8C9)	28-0006	Invitrogen
